# Duramesh registry study: short-term outcomes using mesh suture for abdominal wall closure

**DOI:** 10.3389/fsurg.2023.1321146

**Published:** 2024-01-11

**Authors:** Paige N. Hackenberger, Mehul Mittal, Jeffrey Fronza, Michael Shapiro

**Affiliations:** Department of Surgery, Northwestern Feinberg School of Medicine, Chicago, IL, United States

**Keywords:** hernia, innovation, abdominal wall, incisional hernia, mesh

## Abstract

**Introduction:**

Sutures are flexible linear elements that join tissue and maintain their hold with a surgeon-created knot. Tension at the suture/tissue interface can cut the very tissues that sutures are designed to hold, leading to dehiscence and incisional hernia formation. A new suture design (Duramesh, Mesh Suture Inc., Chicago, IL) was approved for marketing by the United States Food and Drug Administration in September 2022. The multiple filaments of the mesh suture are designed to diffuse tension at the suture/tissue interface thereby limiting pull-through. The macroporosity and hollow core of the mesh suture encourage fibrovascular incorporation for a durable repair. We created the first registry and clinical report of patients undergoing mesh suture implantation to assess its real-world effectiveness.

**Methods:**

A patient registry was created based on institutional implant logs from January to August 2023 at an integrated health-care system. Operative reports were reviewed by the study team to verify use of “Duramesh” by dictation. Retrospective chart review was conducted to evaluate patient and surgical characteristics, follow-up, and short-term outcomes of interest. Results were analyzed using descriptive statistics and Chi-squared analysis with Microsoft Excel and GraphPad Prism.

**Results:**

Three hundred seventy-nine separate implantations by 56 surgeons across 12 (sub) specialties at a university hospital and two community hospitals were performed. Mesh suture was used for treatment of the abdominal wall in 314 cases. Follow-up averaged 80.8 ± 52.4 days. The most common abdominal wall indications were ventral hernia repair (*N* = 97), fascial closure (*N* = 93), abdominal donor site closure from autologous breast reconstruction (*N* = 51), and umbilical hernia repair (*N* = 41). Mesh suture was used in all Centers for Disease Control (CDC) wound classifications, including 92 CDC class 2 or 3 abdominal operations. There were 19 surgical site infections (6.1%) and 37 surgical site events (11.8%).

**Conclusions:**

Short-term registry data demonstrates the wide diversity of surgical disciplines and scenarios in which mesh suture has been used to date. The early adoption of mesh suture into practice highlights that consequences of suture pull-through influence operative decision making. As this is the first interim report of the Duramesh mesh suture registry, follow-up is too short for characterization of long-term durability of abdominal wall closures.

## Introduction

Suturing separated tissues is a core surgical technique often taken for granted. Suture design has remained essentially unchanged since the time of the pharaohs in ancient Egypt (1), with the creation of barbed suture in 2004 only the second suture design innovation since that time (2). With suture, a flexible linear element is passed through two separated tissues and tension is applied, shortening the suture and creating a loop. Suture tension is then maintained by creating a knot. However, even for the most experienced surgeons, the tension needed to approximate tissues is difficult to gauge (3). Too little tension and the tissues do not remain in apposition. Too great a tension, and the suture can slice through the same tissues that they were meant to gently hold (4). The phenomenon of sutures cutting tissues has many synonymous designations including “suture pull-through” and “cheese wiring”; no matter the name, this occurs when the sharp leading edge of the suture applies focused pressure at the suture/tissue interface (STI), and the pressure causes either an abrupt cutting of tissues or a more gradual process of tissue ischemia and scar that remodels over time.

Solving this problem is paramount, as the strain on healthcare resources continues to expand (5–7). Mitigating the problem of concentrated forces causing damage to tissues has been a focus of surgeons of all specialties. In non-hernia scenarios, orthopedic surgeons employ splints and casts to limit tension and range of motion which may otherwise strain critical soft tissue closures. Suture pull-through is the primary culprit in incisional hernia (IH) development which occurs in 24% of sutured laparotomy closures (8). Among those who treat the abdominal wall, solutions are many: the “small-bites” suturing technique (9, 10), the use of planar meshes (11), anterior and posterior component release, preoperative injection of botulinum toxin into the lateral abdominal musculature (12), and minimally-invasive techniques that avoid large abdominal wall incisions entirely. Nevertheless, these alternative and complementary techniques have not yet eliminated IH formation and thus reflect the continuing need for innovation in abdominal wall closure. The “small-bites” technique has a reported 3.3% IH occurrence rate at 1 year (13) and was recently shown to have a 7.6% IH occurrence at 3 years (14). Planar mesh can be used in various planes and as an onlay, inlay, or sublay, with IH formation in 6%–23% of patients (15). Component release(s) and botulinum toxin injections are complementary techniques and can be used alongside many closure methods including the use of mesh suture.

**Figure 1:**
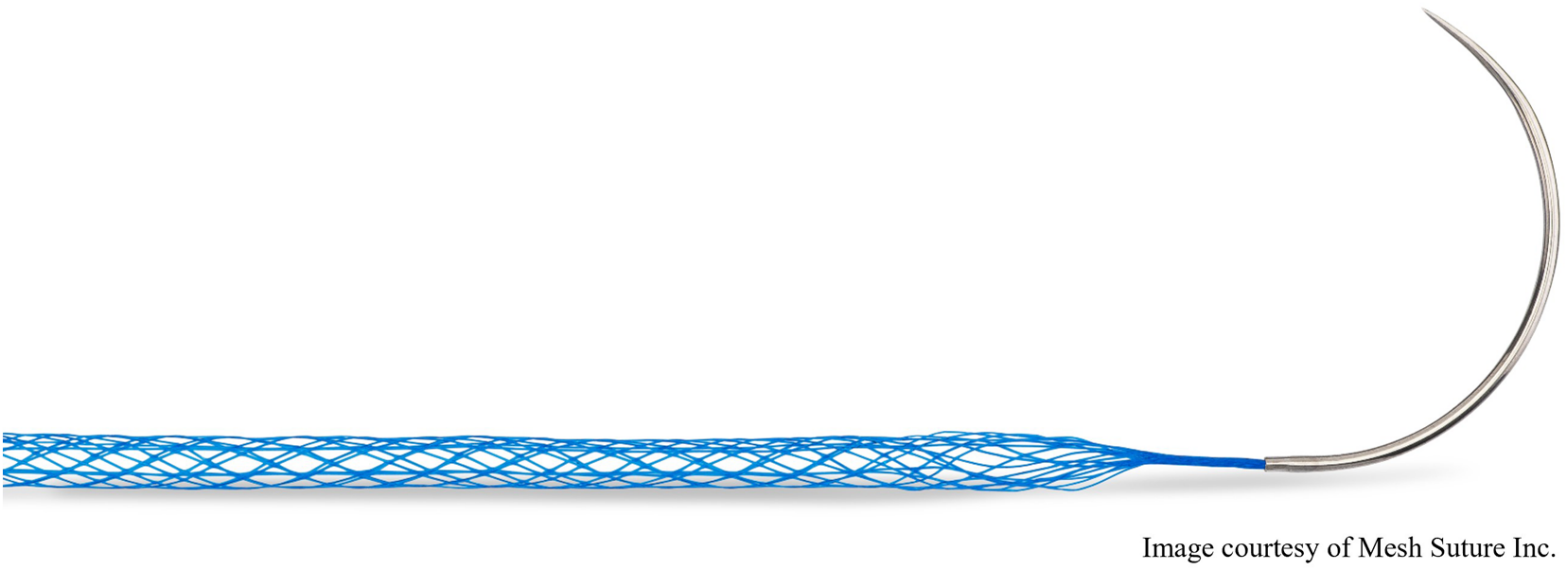
Duramesh mesh suture device (item MSI-301 pictured).

**Figure 2:**
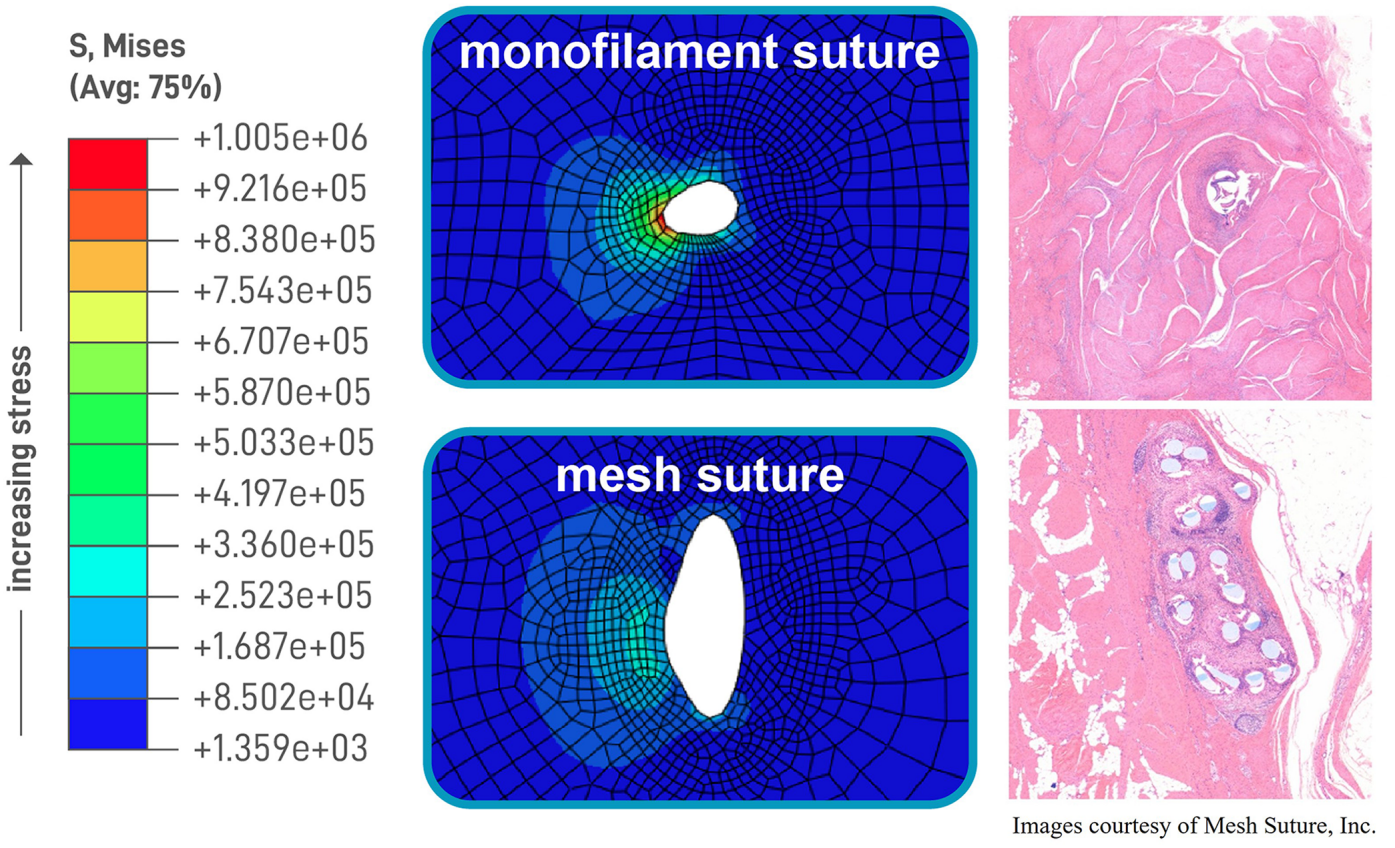
Stress concentration and histologic analysis of traditional suture versus Duramesh.

Mesh suture, the subject of this registry, is a novel suture design created from fine polypropylene filaments that are braided and bonded to create a hollow porous cylinder (Figure 1). While not changing the axial tension along the entire suture, the mesh suture does change shape by flattening like a ribbon upon deployment to create a broad surface area at the suture/tissue interface (STI). The mesh suture filaments distribute the tension and diffuse it at the STI (Figure 2), akin to dulling a knife. By this diffusion mechanism, the mesh suture requires greater force to cut tissue than does standard suture, much like a dull knife requires more force to cut (16). Over time, fibrovascular incorporation of the multiple filaments causes the mesh suture to act as a scar scaffold—employing the natural foreign body reaction to work advantageously in healing (17). Diffusion of tension at the STI combined with fibrovascular incorporation may explain the decreased hernia formation seen in an in-vivo porcine laparotomy model (18).

High-tension surgical closures require that the ultimate tensile strength (UTS) of the repair remains greater than the forces applied in order to prevent acute or chronic suture pull-through and surgical failure (19). For the abdominal wall, durable UTS is achieved with the use of planar meshes that distribute forces over a large surface area (20, 21). A recent innovation has shown how strips of mesh used as suture material avoid the downsides of planar meshes—increased time for placement, large amounts of foreign material, and increased tissue dissection –while maintaining the benefits of force distribution at the STI (22, 23). In these “mesh strip” repairs, a 2 cm wide strip of planar mesh is introduced through either side of the abdominal wall with a sharp instrument and simply tied as a suture. This off-label use of a planar mesh has shown great efficiency and efficacy for both simple and complex abdominal wall closures (19).

While the mesh strip technique is a successful proof of concept for a mesh suture, it is subject to many variables that can alter its efficacy—including accurate strip cutting, strip passing techniques, and even the type of polypropylene mesh available on a hospital's formulary. Furthermore, the added learning curve to perform the mesh strip technique may be insurmountable for surgeons, trainees, and/or operating room staff. These factors limit the generalizability and introduce confounders into analysis of outcomes beyond individual surgeon practices.

Duramesh mesh suture (MSI, Chicago, IL) was approved for marketing by the United States Food and Drug Administration (FDA) in September 2022 as a polyfilament polypropylene suture that distributes forces at the STI and allows fibrovascular incorporation. We created the first registry and clinical report of patients undergoing mesh suture implantation to assess its real-world effectiveness in surgical practice. We review the first 6 months of treated patients to report usage and short-term wound-related outcomes. We will follow this cohort for future commentary on durability of abdominal wall closure.

## Methods

### Registry creation & data collection

Creation of a mesh suture patient registry was approved by the Northwestern University Institutional Review Board (IRB). Patients were identified through institutional implant logs of Duramesh mesh suture from January 23, 2023 to July 31, 2023 at one university-based and two community-based hospitals. Implant logs were consolidated and corresponding operative reports were reviewed by the study team to verify use of Duramesh. The study team has no direct conflicts of interest with Duramesh or with MSI. However, the suture was developed by a member of the Northwestern Feinberg School of Medicine Department of Surgery and the Department received an unrestricted grant of $15,000 which has partially supported the salary of Dr. Hackenberger.

As mesh suture was used as part of standard clinical practice and as decided by their attending surgeon, patients did not give additional informed consent for the use of mesh suture. Surgeons did not receive any incentive or other encouragement to use mesh suture. The Instructions for Use were available to the surgical team with each use of mesh suture. According to the Instructions for Use, the device can be used in both interrupted and running fashion, with placement of each stitch performed slowly to minimize possible tissue damage and surgical bite width and suture spacing is left to specific surgeon assessment based on “years of training, education, experience, and evaluation of tissues” (24). Clinically, the surgeons at our institution report an average of 10 mm bites of fascia and 8 mm travels between bites. A minimum of four alternating throws (2 square knots) for knot security is recommended, with a minimum 3 mm tail after trimming (24). Given the retrospective study design, surgeons performed wound bed preparation, wound edge debridement, and/or wound sterilization based on their unique clinical decision making and did not receive uniform instruction techniques.

Retrospective chart review of the electronic medical record was performed to evaluate patient characteristics, surgical details, and short-term outcomes of interest. Patient characteristics included data pertaining to demographic information, past and current medical history, past surgical history, and hernia history. Determination of patient's pre-operative hernia status was abstracted from documentation of one or more of the following: findings from abdominal physical exam performed by a medical professional, abdominal CT scan, or operative report description of the abdominal wall. No new data entries for follow-up were added after September 30, 2023.

Surgical details included service line/specialty, indication for mesh suture use, and Centers for Disease Control (CDC) wound classification as categorized by the surgical team at the time of the procedure. Mesh suture implant details including anatomic location of mesh suture implantation, suture size, needle type, and number of implants used were collected for each operation. Patient charts were reviewed for documented follow-ups, with outcome collection only stopped for patients with re-operation through the mesh suture repair or Duramesh removal for any reason.

### Outcomes assessment

The primary outcome for this interim report was incidence of surgical site infections (SSI) and surgical site events (SSE) in abdominal wall treatments per definitions by Majumder et al. (Table 1) and/or incisional hernia development or recurrence after closure with Duramesh (25). SSI include superficial, deep, and/or organ/space infections. SSE include seroma, hematoma, soft tissue breakdown, fascial dehiscence, cellulitis, suture granuloma, chronic draining sinus, and/or enterocutaneous fistula formation. Abdominal wall indications included fascial closure, ventral hernia repair, donor site closure for deep inferior epigastric perforator (DIEP) flap breast reconstruction, umbilical hernia repair, and miscellaneous cases including parastomal hernia repair, total abdominal wall reconstruction, and rectus diastasis plication.

**Table 1:** Definitions of surgical site infections (SSI) and surgical site events (SSE).

	Definition
SSI	Events occurring within 90 days of hernia repair or up to 1 year for deep and organ/ space SSIs with presence of implant
Superficial	Infection involving skin or subcutaneous tissue along with 1 + of the following: purulent drainage, organisms isolated from fluid/tissue, 1 sign of inflammation (pain/tenderness, induration, erythema, local warmth), deliberate wound opening by surgeon, or surgeon declaration
Deep	Infection involves deep soft tissues (fascia and/or muscle) with 1 + of the following: purulent drainage, fascial dehiscence with signs of inflammation, deliberate fascial separation by surgeon, deep abscess identified by direct examination, reoperation or radiologic verification, or surgeon declaration
Organ/space	Infection involves anatomic structures not opened or manipulated by operation or peritoneal cavity with 1 + of the following: purulent drainage from drain or incision into organ/space, organisms isolated by aseptic culture, identification of abscess by direct identification, reoperation, or radiologic verification, or diagnosis by surgeon declaration
SSE	Events occurring within 90 days of hernia repair
Seroma	Collection of serous fluid in abdominal wall that is either symptomatic (causes pain/discomfort) or requires intervention
Hematoma	Collection of blood in the abdominal wall that is either symptomatic (causes pain/discomfort) or requires intervention
Soft tissue breakdown	Skin and/or adipose tissue breakdown requiring debridement or packing. Does not include fascial dehiscence
Fascial dehiscence	Fascial separation without evidence of infection or inflammation requiring clinical intervention
Cellulitis	Erythema of skin or subcutaneous connective tissue that does not involve the surgical site but requires treatment with antibiotics
Suture granuloma	Localized inflammatory reaction in response to retained suture material without evidence of infection requiring intervention
Chronic draining sinus	Sinus tract in abdominal wall draining serous or fibrinous fluid without evidence of gross purulence
Enterocutaneous fistula	Connection from the gastrointestinal tract to the skin with spillage of enteric contents

Table from Majumder et al. (25).

Secondary outcomes of interest included index case length of stay and sequelae of documented adverse outcomes as defined above. Patients with a documented SSI and/or SSE were evaluated for index surgery related readmission(s), and/or reoperation(s) and associated timing of these visits.

### Data analysis

Standard descriptive summary statistics were used for patient characteristics, surgical details, and outcomes of interest. Continuous variables were reported as means with standard deviations and categorical variables were reported as proportions. Data were stratified by occurrence of SSI and SSE, presence of pre-operative hernia, and Duramesh indication. Groups were compared using Chi-squared test or Fisher exact test for categorical variables and unpaired *t*-tests for continuous variables. A *p*-value of <0.05 was considered significant. Data were managed and analyzed using Microsoft Excel (Redmond, WA) and GraphPad Prism (Boston, MA).

## Results

### Patient, provider, and surgical details

Three hundred seventy-nine patients were implanted with mesh suture at Northwestern Memorial Hospital, Northwestern Lake Forest Hospital, and Northwestern Kishwaukee Hospital from January 23, 2023 to July 30, 2023. As outlined in Table 2, patients were on average 57.3 ± 13.9 years old, 60% (*N* = 226) were female, and 77% (*N* = 290) were white/Caucasian. Average body mass index (BMI) was 30.1 ± 7.0 kg/m^2^. Prevalence of diabetes 19% (*N* = 70), hypertension 46% (*N* = 176), chronic obstructive pulmonary disease (COPD) 6% (*N* = 21), and cancer history 36% (*N* = 136) were recorded. 13% (*N* = 50) of patients were active smokers.

**Table 2:** Patient details for mesh suture uses (*N* = 379).

Patient demographics	
Average age (years)	57.3 ± 13.9
Female	60% (226)
White/Caucasian	77% (290)
Average BMI (kg/m^2)	30.1 ± 7.0
Diabetes	19% (70)
HTN	46% (176)
COPD	6% (21)
Cancer	36% (136)
Smoking	
Active (<4 weeks)	13% (50)
Former (>4 weeks)	27% (104)

**Figure 3:**
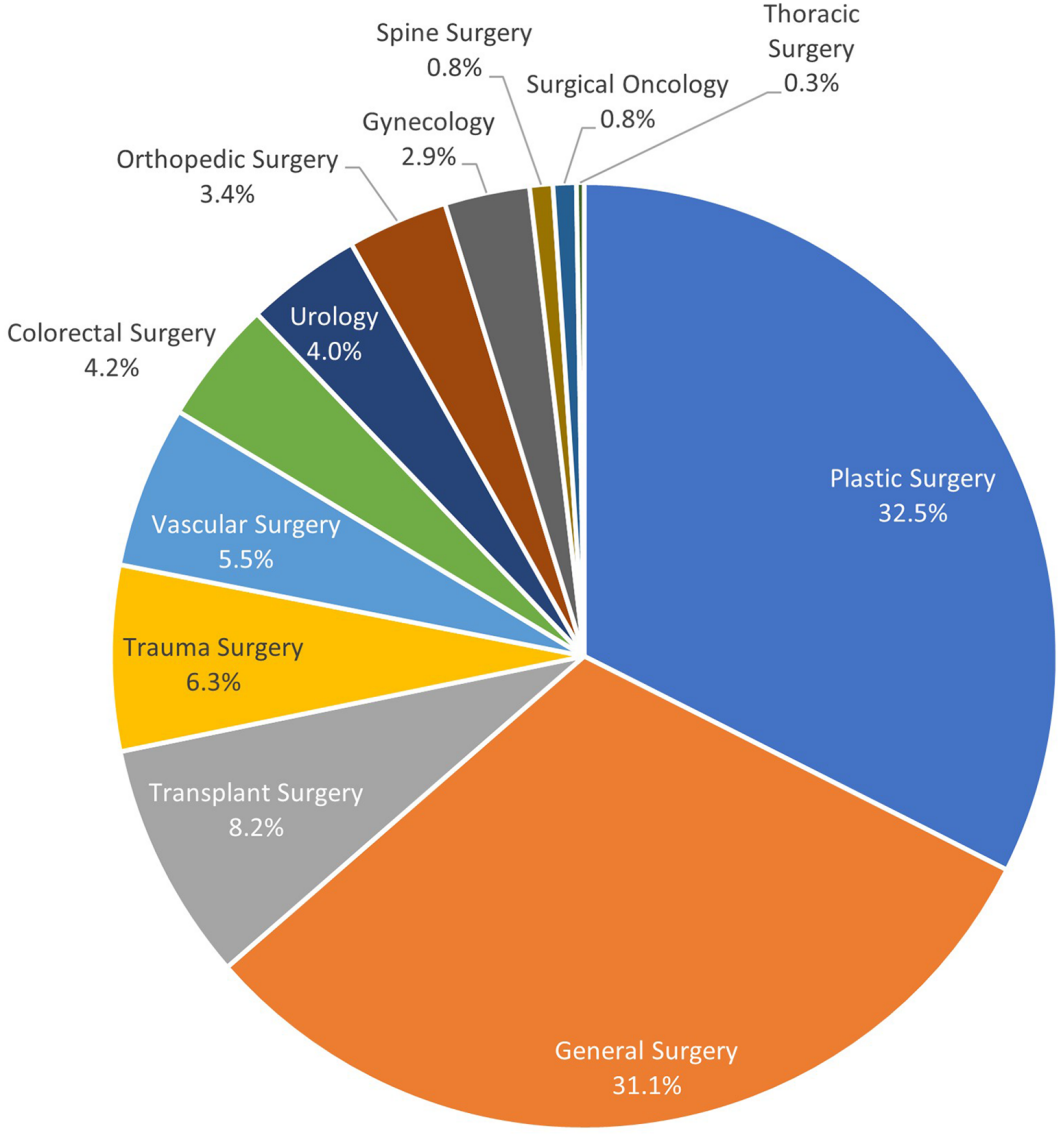
Mesh suture use by surgeon specialty.

**Figure 4:**
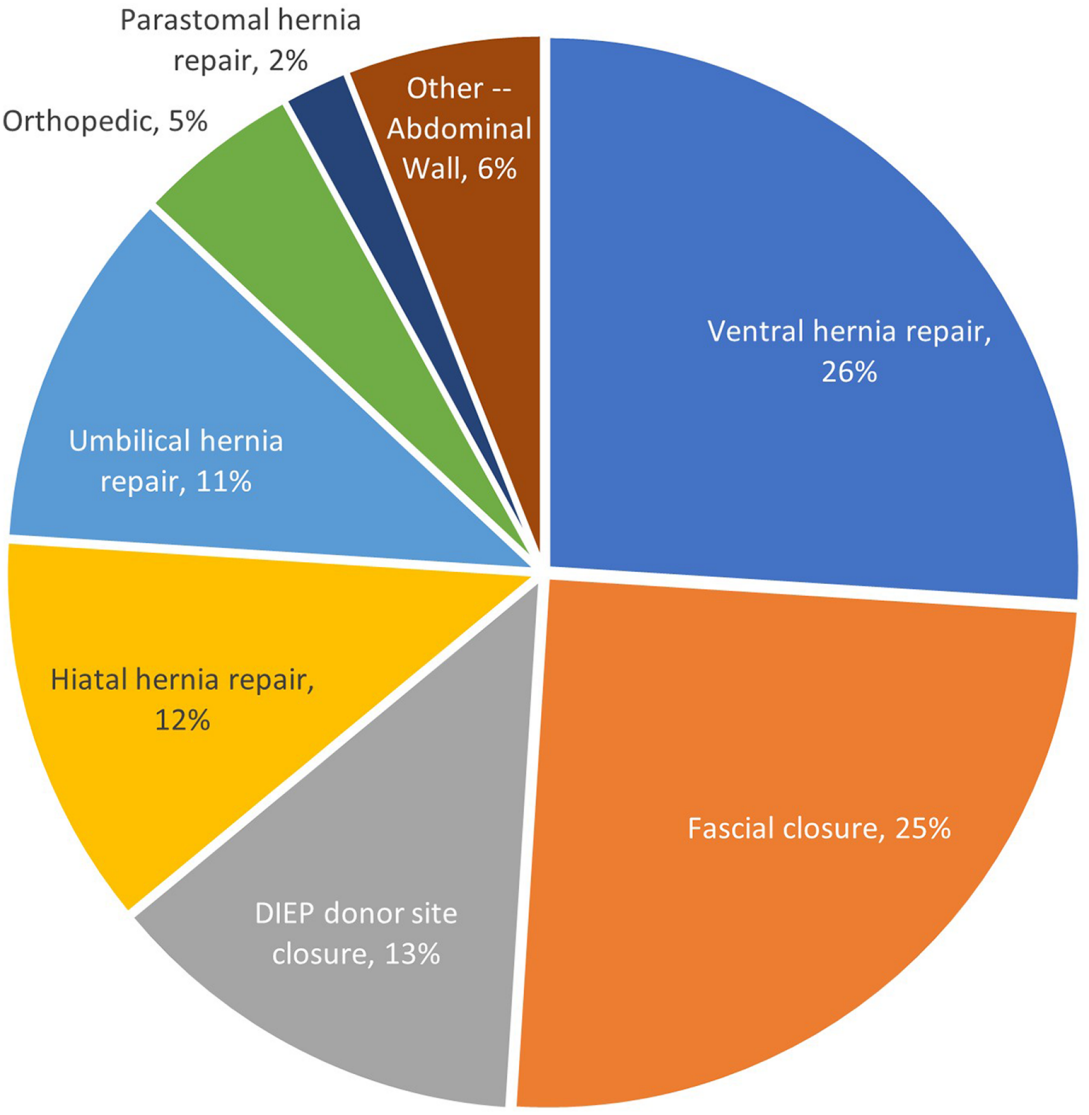
Mesh suture use by indication.

Fifty-six surgeons from 12 disciplines used Duramesh in these 379 cases (Figure 3). Three hundred fourteen instances of use were for abdominal wall implantations, the remainder were used for mostly hiatal hernia or orthopedic indications. Of the patients with abdominal wall implantations, 48% (*N* = 152) had evidence of a pre-existing hernia, and 25% (*N* = 80) had a recurrent hernia after a previous, non-mesh suture repair. Figure 4 outlines the various indications for Duramesh use. The 5 most common use cases for mesh suture were ventral hernia repair (26%, *N* = 97), fascial closure at the time of laparotomy (25%, *N* = 93), donor site closure for DIEP flap breast reconstruction (13%, *N* = 51), hiatal hernia repair (12%, *N* = 46), and umbilical hernia repair (11%, *N* = 41). Frequency of mesh suture item type (including suture size and needle size) and average number of mesh sutures for common surgical indications is further detailed in Appendix A.

### Abdominal wall outcomes

Of the 314 abdominal wall cases, 83.8% (*N* = 263) had no complications. Average follow up duration was 81.9 ± 52.6 days. Division of cases by Centers for Disease Control (CDC) wound classifications were as follows: 65.9% (*N* = 207) clean, 21.0% (*N* = 66) clean-contaminated, 8.3% (*N* = 26) contaminated, and 4.8% (*N* = 15) dirty/infected (Table 3).

**Table 3:** Outcomes by abdominal wall indication (*N* = 314).

	All abdominal wall indications (*N* = 314)	Ventral hernia repair (*N* = 97)	Fascial closure (*N* = 93)	DIEP flap donor site closure (*N* = 51)	Umbilical hernia repair (*N* = 41)	Other abdominal wall (*N* = 32)
CDC wound classification						
1: Clean	65.9% (207)	69.1% (67)	36.6% (34)	100% (51)	85.4% (35)	62.5% (20)
2: Clean-contaminated	21.0% (66)	18.6% (18)	39.8% (37)	–	14.6% (6)	15.6% (5)
3: Contaminated	8.3% (26)	12.4% (12)	9.7% (9)	–	–	15.6% (5)
4: Dirty/infected	4.8% (15)	–	14.0% (13)	–	–	6.3% (2)
Patients without SSI or SSE	83.8% (263)	76.3% (74)	90.3% (84)	82.4% (42)	87.8% (36)	84.4% (27)
Surgical site infections (SSI)	6.1% (19)					
Superficial	2.5% (8)	3.1% (3)	2.2% (2)	2.0% (1)	4.9% (2)	–
Deep	1.0% (3)	3.1% (3)	–	–	–	–
Organ/space	2.9% (9)	5.2% (5)	3.2% (3)	–	–	3.1% (1)
Surgical site events (SSE)	11.8% (37)					
Seroma	4.5% (14)	8.2% (8)	1.1% (1)	5.9% (3)	–	6.3% (2)
Hematoma	1.0% (3)	1.0% (1)	–	–	2.4% (1)	3.1% (1)
Soft tissue breakdown	3.5% (11)	2.1% (2)	1.1% (1)	11.8% (6)	2.4% (1)	3.1% (1)
Fascial dehiscence	1.6% (5)	2.1% (2)	2.2% (2)	–	2.4% (1)	–
Cellulitis	0.3% (1)	1.0% (1)	–	–	–	–
Suture granuloma	1.0% (3)	2.1% (2)	–	–	2.4% (1)	–
Chronic draining sinus	0.3% (1)	–	1.1% (1)	–	–	–
Enterocutaneous fistula	0.3% (1)	1.0% (1)	–	–	–	–
Hernia development/recurrence	0.6% (2)	1.0% (1)	1.1% (1)	–	–	–

DIEP, Deep inferior epigastric perforator.

One patient may have more than one recorded outcome such that sum of SSI and SSE equals more than total number of patients with complications overall.

Notable outcomes included surgical site infections (SSI), surgical site events (SSE), and/or hernia development/recurrence. Importantly, patients may have more than one recorded SSI and/or SSE by definition. Patients with any complication were also reviewed for hospital readmissions and/or reoperations of any etiology as well as in relation to the mesh suture surgery. Our study reports an overall SSI of 6.1% (*N* = 19) and SSE of 11.8% (*N* = 37) across 314 abdominal wall closures (Table 3). SSI are further subclassified with 2.5% (*N* = 8) superficial, 1.0% (*N* = 3) deep, and 2.9% (*N* = 9) organ/space infections. SSE are also further subclassified with 4.5% (*N* = 14) seroma, 1.0% (*N* = 3) hematoma, 3.5% (*N* = 11) soft tissue breakdown, 1.6% (*N* = 5) fascial dehiscence, 0.3% (*N* = 1) cellulitis, 1.0% (*N* = 3) suture granuloma, 0.3% (*N* = 1) chronic draining sinus, and 0.3% (*N* = 1) enterocutaneous fistula formation. There was a 0.6% (*N* = 2) incidence of hernia development/recurrence. These outcomes are presented in tabular form in Table 3.

A contingency table was designed to assess possible effects of patient preoperative characteristics on incidence of SSI and SSE. Groups were overall comparable in demographic characteristics except for CDC wound classification (*p* = 0.021) and preoperative hernia presence (*p* = 0.031) distributions in relation to development of SSI (Appendix B).

Significant adverse events were deemed by the study team to include: organ/space infection, fascial dehiscence, chronic draining sinus, enterocutaneous fistula, and/or hernia development/recurrence. These patients underwent additional review for the context of their complication(s). These are further outlined in Appendix C.

### Subgroup analysis

Abdominal wall patients were further grouped for analysis. Groups were compared for incidence of complications, index case length of inpatient stay, follow-up duration, and details of readmissions and reoperations in patients with documented complications. Groups were compared using Chi-squared test or Fisher exact test for categorical variables and unpaired *t*-tests for continuous variables.

The first pairing (Table 4) was between patients without preoperative hernias (*N* = 162) and those with a preoperative hernia (*N* = 152). Index case length of inpatient stay was 3.9 ± 5.4 days in the no hernia group compared to 6.6 ± 8.7 days in the hernia group (*p* = <0.001). The only other significant difference was in follow-up duration with an average of 88.5 ± 53.2 days in the no hernia group compared to 74.9 ± 51.2 days in the hernia group (*p* = 0.022).

**Table 4:** Summary of surgical outcomes for abdominal wall patients with and without preoperative hernia (*N* = 314).

Outcome	No hernia (*N* = 162)	With hernia (*N* = 152)	*p*-value
Patients without complications	87.0% (141)	80.3% (122)	0.1258
Surgical complication*			
Superficial infection	1.9% (3)	3.3% (5)	0.4902
Deep infection	–	2.0% (3)	0.1123
Organ/space infection	1.2% (2)	4.6% (7)	0.0951
Seroma	3.7% (6)	5.3% (8)	0.5897
Hematoma	–	2.0% (3)	0.1123
Soft tissue breakdown	4.9% (8)	2.0% (3)	0.2209
Fascial dehiscence	1.2% (2)	2.0% (3)	0.6760
Cellulitis	–	0.7% (1)	0.4841
Suture granuloma	0.6% (1)	1.3% (2)	0.6121
Chronic draining sinus	0.6% (1)	–	>0.999
Enterocutaneous fistula	–	0.7% (1)	0.4841
Hernia development/recurrence	0.6% (1)	0.7% (1)	>0.999
Index case LOS (days)	6.6 ± 8.7	3.9 ± 5.4	0.0003
Follow-up duration (days)	88.5 ± 53.2	74.9 ± 51.2	0.0218
Patients with complications	21	30	
Number of readmissions	5	10	0.5431
Readmissions related to abdominal closure	3	7	0.4949
Number of reoperations	7	11	>0.999
Reoperations related to abdominal closure	4	7	>0.999

LOS, length of stay.

Abdominal wall indications include: fascial closure, ventral hernia repair, DIEP flap donor site closure, umbilical hernia repair, etc.

* One patient may have more than one recorded outcome such that sum of SSI and SSE equals more than total number of patients with complications overall.

The second pairing (Table 5) looked at patients with CDC class 2 (clean-contaminated) or 3 (contaminated) closures indicated for either fascial closure (*N* = 46) or ventral hernia repair (*N* = 30). Percent of patients without complications was significantly higher in the fascial closure group (91.3%) than the ventral hernia repair group (66.7%; *p* = 0.013).

**Table 5:** Summary of surgical outcomes for fascial closures and ventral hernia repairs in CDC class 2 & 3 fields.

Outcome	CDC 2 & 3 fascial closure (*N* = 46)	CDC 2 & 3 ventral hernia repair (*N* = 30)	*p*-value
Patients without complications	91.3% (42)	66.7% (20)	0.0133
Surgical complication*			
Superficial infection	–	6.7% (2)	0.1526
Deep infection	–	6.7% (2)	0.1526
Organ/space infection	4.3% (2)	13.3% (4)	0.4103
Seroma	2.2% (1)	6.7% (2)	0.5583
Hematoma	–	–	–
Soft tissue breakdown	–	6.7% (2)	0.1526
Fascial dehiscence	4.3% (2)	6.7% (2)	0.6450
Cellulitis	–	3.3% (1)	0.3947
Suture granuloma	–	–	–
Chronic draining sinus	–	–	–
Enterocutaneous fistula	–	–	–
Hernia development/recurrence	–	–	–
Index case LOS (days)	7.6 ± 6.7	6.9 ± 5.6	0.6240
Follow-up duration (days)	77.0 ± 50.0	85.2 ± 52.2	0.4988
Patients with complications	4	10	
Number of readmissions	3	6	>0.999
Readmissions related to abdominal closure	2	5	>0.999
Number of reoperations	4	4	0.0849
Reoperations related to abdominal closure	2	3	0.5804

LOS, length of stay.

* One patient may have more than one recorded outcome such that sum of SSI and SSE equals more than total number of patients with complications overall.

## Discussion

This report is the first to outline the breadth of use cases in real-world context following FDA approval of Duramesh in late 2022. Furthermore, we quantify short-term outcomes of interest, with a focus on 314 cases where Duramesh use was related to treatment of the abdominal wall. The widespread adoption of mesh suture by 56 surgeons across 12 surgical specialties speaks to the general understanding of the damaging effects of suture pull-through and highlights the need for a better surgical solution.

### Safety and efficacy

With any implant, there is concern that the foreign body response can lead to unexpected clinical outcomes. In treatment of the abdominal wall, planar mesh has been associated with infection, adhesions, and chronic draining sinus formation among other conditions (26–29). The data from this registry study supports the safe and efficacious use of mesh suture for abdominal wall closures. Our study reports an overall SSI of 6.1% (*N* = 19) and SSE of 11.8% (*N* = 37) across 314 abdominal wall closures. Incidence of SSI and SSE varied by abdominal wall indication, with fascial closures having the lowest percentage of patients with complications (9.7%) and ventral hernia repairs representing the highest (23.7%). There were no bowel obstructions due to a mesh suture adhesion.

These data compare favorably with complication rates described in the literature from other large, diverse, academic centers who report outcomes after elective abdominal wall closures in patients of similar demographics (30, 31). Our overall SSI rate was low (6.1%), however this may relate in part to our inclusion of clean umbilical hernia and DIEP flap closures. Contaminated abdominal wall closures were well represented in our study and reflect the reality of many abdominal wall surgeons’ practices.

Forty-six patients with clean-contaminated (CDC 2) and contaminated (CDC 3) laparotomy incisions were closed with Duramesh and had an overall complication rate of 8.7%, with only 2 readmission and 2 reoperations relating to the abdominal wall closure (Table 5). These patients’ early complication rate of 8.7% can be compared to a 13.5% early complication rate in a cohort of 12,373 patients undergoing laparotomy closure at the University of Pennsylvania (30), and a 25% infection rate in a study of 696 patients undergoing laparotomy closure from Technische Universitat of Dresden, Germany (31).

Thirty patients in our study underwent CDC 2 or 3 mesh suture incisional hernia repairs with an overall complication rate of 33.3%. This compares to reported surgical site complication rates of 31%–52% for retrorectus polypropylene mesh, 28% for absorbable synthetic mesh, and 66% for bioprosthetic mesh (32). In our experience, these other treatments of permanent, absorbable, and/or bioprosthetic meshes require significant tissue plane dissection and increased operative time when compared to the simplicity of a mesh suture closure. In addition, mesh suture can be easily located beneath the skin incision if a need for removal arises, and preserves the retrorectus space as a ‘lifeboat’ reconstructive option should it ever be needed. When subgroup analysis of the full abdominal wall cohort was conducted, CDC wound classification distributions were significantly different between patients who did and did not develop SSI. On further evaluation of outcomes between fascial closures and ventral hernia repairs in CDC 2 and 3 wound classes, significantly more patients developed complications in the ventral hernia repair group.

Hernia development or recurrence after abdominal wall closure is a critical outcome that denotes the durability of a repair technique. Currently, follow-up duration in our patient registry is too short to reasonably comment on the incidence of this event as one year is typically regarded as a minimum amount of time required to assess this outcome (33). While there is no upper limit for the amount of time to follow-up patients for development of a hernia, most incisional hernias will occur within 2 years after repair (34). There were two early hernia recurrences in our registry cohort that represent this outcome on a short-term timeline (average follow-up of 80.8 days). Use of surgical risk stratification for IH development as described by Fischer et al. has shown relevance in patient selection, preoperative optimization, and surgical approach to reduce complications and contain costs (30). Through careful selection of high- or extreme-risk patients, use of prophylactic onlay mesh is applied with discernment to reduce postoperative IH development in those with the greatest predicted risk (30). Whether data will support preferential use of Duramesh at particular risk levels or replace the need for prophylactic onlay mesh in certain populations remains to be seen at this time.

### Notable outcomes

**Figure 5:**
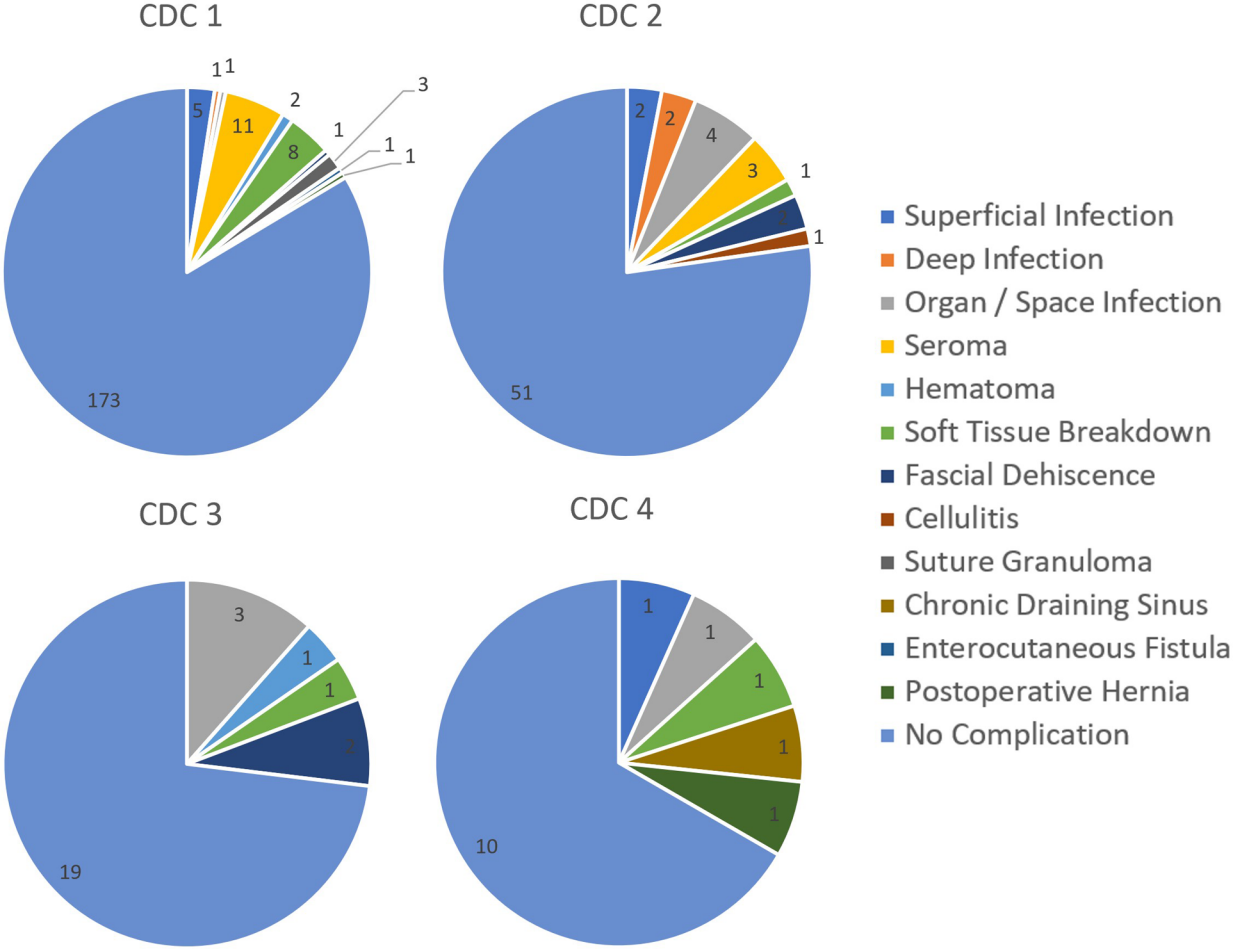
Surgical outcomes by CDC wound classification.

Fifteen patients underwent off-label implantation of mesh suture in CDC class 4 (dirty/infected) fields. These may have occurred in scenarios where the surgeon felt the benefits of using mesh suture outweighed the expected risks. In these patients, SSI and SSE incidence were each 13% and significant adverse events occurred in 20% of patients. This is notably higher than the incidence of complications compared to any other CDC classification (Figure 5). One patient who had implantation in a CDC 4 wound went on to develop a chronic draining sinus. A Cochrane review has shown that use of absorbable sutures can reduce the risk of sinus or fistula tract formation compared to permanent material (such as that used in mesh suture) (35). However, despite the polyfilament mesh outer design with increased surface area in comparison to a standard suture, this single occurrence within the larger cohort (0.3%) is considerably lower than the literature rate of 3.5% for other permanent sutures, although longer follow-up may yield a higher final percentage (35).

Fascial dehiscence occurred in 5 patients. One occurred in an actively smoking urologic cancer patient who underwent robotic cystectomy and ileal conduit creation, and subsequently developed a culture-positive fascial infection. One occurred in an actively smoking, diabetic sarcoma patient with a CDC class 4 surgical field (off-label use) due to active sepsis and bowel leak. One occurred in an immunosuppressed transplant patient with uncontrolled diabetes (HbA1c >8%) and a recent fascial dehiscence earlier during the same admission. Finally, two occurred due to knot slippage. One was in a patient who underwent uneventful umbilical hernia repair secured with a 6-throw “granny” knot. The other was in a radiated urologic cancer patient who underwent radical cystoprostatectomy followed by fascial closure secured with a 3-throw knot. Nevertheless, we report an overall fascial dehiscence rate of 1.6%; lower than a recent Cochrane database report of 3.3% for permanent suture (35) and for the German university study with 696 patients (7.6%) (31).

These instances highlight how patient characteristics, applied surgical technique, and judgment can affect outcomes. According to the Duramesh Instructions for Use, mesh suture is contraindicated in CDC class 4 wounds (24). Furthermore, proper knot tying is required for optimal performance, with the product recommendation being “at least 4 alternating throws”, an explicit discouragement of knots that fail to alternate direction (“granny knots”), and “crimping” of the knot with an extra amount of force while tying at the end of each throw (24). As dehiscence is a serious complication, surgeons at our institution report placing one or two additional throws for added knot security. Divergence from any device's intended use may lead to higher likelihood of complications and materials regarding proper handling should be studied with caution.

### Limitations

This study is limited by follow-up duration and possible confounding by indication. As this is the first study to report outcomes from use of Duramesh in patients since FDA approval, only short-term outcomes were able to be queried. The formation of a registry will allow us to continually report on outcomes as follow-up duration continues. Nevertheless, we find that these data support the versatility and breadth of applications of mesh suture in the surgeon's armamentarium while identifying incidence of short-term notable outcomes to demonstrate how it can best be used.

These data do not yet satisfy the question for whom mesh suture is best indicated, but rather serve as a map of real-time use patterns of this new medical device. As this was a retrospective review, patients were not prospectively enrolled or randomized for use of mesh suture, and therefore the population of our registry may be confounded by uncategorized variables. Randomized control trials are underway to best highlight more specific indications and outcomes for those undergoing implantation with mesh suture. As our cohort grows, we plan to add a control group via propensity matching to allow for accurate comparisons and reduce the influence of any outcome mediators. This will allow us to report outcome metrics in comparison to an equivalent cohort and assess for differences between closure techniques and resultant complication profiles that may alter the risk-benefit assessment in certain populations.

Lastly, no cost data was collected as part of this study, however costs of the device are typically country and/or insurance provider specific and can vary widely. At our institution, Duramesh is more expensive than specialty sutures, and less expensive than specialty meshes, absorbable meshes, and bioprosthetic meshes. A cost analysis of mesh suture is warranted in the future to ascertain the economic impact of this device compared to alternatives.

## Conclusion

Short-term registry data demonstrates the wide diversity of surgical disciplines and scenarios in which mesh suture has been used to date. The early adoption of mesh suture into practice highlights that consequences of suture pull-through influence operative decision making. In treatment of the abdominal wall, data are promising, with low incidence of surgical site infections and surgical site events. As with any new device, it is imperative that adopters carefully review the instructions for proper use(s) to optimize outcomes. As this is the first interim report of the Duramesh mesh suture registry, follow-up is too short for characterization of long-term durability of abdominal wall closures.

## Data Availability

The datasets presented in this article are not readily available because the registry and corresponding dataset are not publicly available. Requests to access the datasets should be directed to PH, paige.hackenberger@nm.org.

## Appendix A

**Table:** Duramesh item used by indication.

	Ventral hernia repair	Fascial closure	DIEP flap donor site closure	Umbilical hernia repair	Other abdominal wall	Hiatal hernia repair	Orthopedic
Item Master: suture size, needle size							
MSI-100: 2-0, small (DR20)	–	–	2	–			5
MSI-200: 0, small (HR22)	8	–	3	7	1	46	3
MSI-201: 0, large (HR48)	6	15	–	5	1	–	2
MSI-300: 1, small (HR26)	16	12	4	23	11	–	2
MSI-301: 1, large (HR48)	36	49	39	7	6	–	–
MSI-500: 2, small (HR26)	10	3	2	2	6	–	2
MSI-501: 2, large (HR50)	29	18	4	3	10	–	5
Duramesh used per case (mean)	1.63 ± 0.82	1.80 ± 0.73	1.38 ± 0.53	1.33 ± 0.57	1.47 ± 0.67	1.67 ± 0.87	1.61 ± 0.85

## Appendix B

**Table:** Demographics, operative factors by surgical site infection (SSI) and surgical site events (SSE) in abdominal uses of Duramesh.

	Surgical site infection (SSI)					Surgical site event (SSE)				
	No SSI (*N* = 295, 93.9%)		SSI (*N* = 19, 6.1%)		*p-value*	No SSE (*N* = 277, 88.2%)		SSE (*N* = 37, 11.8%)		*p-value*
	*N*	%	*N*	%	*χ²*			*N*	%	*χ²*
Age at operation (year, mean ± SD)	56.8 ± 13.7		58.6 ± 7.6		0.1055	56.6 ± 13.4		59.7 ± 12.9		0.2097
<50	98	33.2%	4	21.1%	4.4980	94	33.9%	8	21.6%	3.1240
50–64	100	33.9%	11	57.9%		98	35.4%	13	35.1%	
65+	97	32.9%	4	21.1%		85	30.7%	16	43.2%	
Legal sex					*0.1471					*0.5933
Female	179	60.7%	8	42.1%		163	58.8%	24	64.9%	
Male	116	39.3%	11	57.9%		114	41.2%	13	35.1%	
BMI (kg/m², mean ± SD)	30.1 ± 7.2		32.0 ± 8.4		*0.8174	30.0 ± 7.3		31.5 ± 7.0		*0.1588
<30	163	55.3%	10	52.6%		157	56.7%	16	43.2%	
≥30	132	44.7%	9	47.4%		120	43.3%	21	56.8%	
Smoking status					*0.3195					*0.8005
Active	40	13.6%	4	21.1%		40	14.4%	4	10.8%	
Not active	255	86.4%	15	78.9%		237	85.6%	33	89.2%	
Diabetes mellitus					*0.7710					*0.5060
Yes	57	19.3%	4	21.1%		52	18.8%	9	24.3%	
No	238	80.7%	15	78.9%		225	81.2%	28	75.7%	
HTN				*>0.999					*0.8629	
Yes	140	47.5%	9	47.4%		132	47.7%	17	45.9%	
No	155	52.5%	10	52.6%		145	52.3%	20	54.1%	
COPD					*0.6129					*0.4556
Yes	18	6.1%	0	0.0%		15	5.4%	3	8.1%	
No	277	93.9%	19	100.0%		262	94.6%	34	91.9%	
Cancer					*0.3330					*0.7207
Yes	119	40.3%	5	26.3%		108	39.0%	16	43.2%	
No	176	59.7%	14	73.7%		169	61.0%	21	56.8%	
CDC wound classification					0.0207					0.9390
1	200	67.8%	7	36.8%	7.7510	182	65.7%	25	67.6%	0.1258
2 & 3	82	27.8%	10	52.6%		82	29.6%	10	27.0%	
4	13	4.4%	2	10.5%		13	4.7%	2	5.4%	
Preoperative hernia					*0.0314					*0.4883
Yes	138	46.8%	14	73.7%		132	47.7%	20	54.1%	
No	157	53.2%	5	26.3%		145	52.3%	17	45.9%	

* Denotes use of Fisher's exact test.

## Appendix C

**Table:** Abdominal Wall adverse events descriptive outcomes.

Adverse event type	CDC wound classification	Description of outcome(s)
Deep infection	1	Diabetic with recurrent ventral hernia closed with Duramesh. Developed symptomatic abscess on POD 17 which required IR drainage. **Duramesh intact.**
Deep infection Fascial dehiscence	2	Active smoker with complex urologic history and recent bladder cancer diagnosis who had Duramesh fascial closure after oncologic surgery. Developed a culture-positive fascial infection and eventual fascial dehiscence on POD 8 requiring reoperation. **Duramesh removed.**
Deep infection Organ/space infection	2	Diabetic, former smoker on transplant immunosuppression who had a fascial infection and fascial dehiscence repaired with Duramesh. Developed a repeat perihepatic abscess and abdominal wall abscess requiring packing of wound and IV antibiotics. **Duramesh intact.**
Organ/space infection	2	Rectourethral fistula repair complicated by early incisional hernia repaired with Duramesh. Developed symptomatic pelvic abscess on POD 24 requiring IR drainage, admission for IV antibiotics, and local wound care. **Duramesh intact.**
	2	Former smoker on Crohn's immunosuppression who underwent bowel surgery and fascial closure with Duramesh. Developed bowel leak and corresponding abscess formation requiring reoperation on POD3. **Reclosed with Duramesh.**
	2	Former smoker who underwent ostomy reversal and ostomy site repair with Duramesh. Developed bowel leak and corresponding abscess formation requiring reoperation on POD5. **Duramesh removed.**
	3	Enterocutaneous fistula takedown and mesh excision with Duramesh closure of midline hernia. Developed symptomatic small bowel fistulae and sinus tract formation requiring readmission without operative intervention. **Duramesh intact.**
	3	Emergent laparotomy for incarcerated ventral hernia requiring bowel resections in a patient with BMI 54, intially left open. Fascia closed with Duramesh on POD1. Developed bowel leaks and frank contamination requiring reoperation on POD5. **Duramesh removed** and abdomen left open.
	4	Exploratory laparotomy fascial closure with Duramesh. Developed pancreatic leak and corresponding abscess formation followed by hemodynamic instability and cardiopulmonary arrest leading to death. **Duramesh intact.**
Organ/space infection Fascial dehiscence	4	Active smoker with diabetes underwent retroperitoneal tumor excision complicated by a bowel leak requiring reoperation and fascial closure with Duramesh. Developed persistent intra-abdominal infections leading to wound breakdown and controlled fascial dehiscence (preventing evisceration) on POD16 requiring reoperation. **Duramesh removed.**
Organ/space infection Enterocutaneous fistula	1	Complex abdominal history including prior enterocutaneous fistula. Underwent excision of old mesh and ventral hernia repair with Duramesh. Developed intra-abdominal abscesses 2 months postop for which she underwent reoperation at an outside facility followed by concern for enterocutaneous fistula formation and need for TPN. **Duramesh intact** (except for 2cm length where I&D performed).
Fascial dehiscence	1	Umbilical hernia repair with Duramesh. Developed fascial dehiscence and SBO on POD2 requiring reoperation and placement of planar mesh. **Duramesh removed.**
	2	Former smoker, with neoadjuvent radiation who underwent oncologic procedure and fascial closure with Duramesh. Developed fascial dehiscence from presumed knot unraveling on POD8 requiring reoperation. 3 knots used in Duramesh per surgeon report. **Duramesh removed.**
	3	Diabetic (HbA1c >8%) on transplant immunosuppression who underwent closure of a fascial dehiscence with Duramesh. Developed repeat fascial dehiscence on POD24. Reclosed with mesh strip technique. **Duramesh removed.**
Chronic draining sinus Postoperative hernia	4	Complex abdominal history including repeated bowel leaks requiring washout and fascial closure with Duramesh. Skin left to to heal by secondary intention. Skin healed initially but 4 months postop developed new drainage for which underlying Duramesh knots were excised during an elective ostomy takedown. At time of ostomy takedown, patient noted to have asymptomatic, small epigastric hernia at site of prior fascial closure. **Duramesh removed.**
Postoperative hernia	1	Recurrent umbilical hernia repaired with Duramesh. Developed symptomatic recurrence 2 months postop for which he underwent reoperation and placement of planar mesh. **Duramesh removed.**
